# Single‐Atom Nano‐Islands: Unlocking New Horizons in Catalytic Activity and Stability

**DOI:** 10.1002/adma.202503361

**Published:** 2025-06-18

**Authors:** Peng Wang, Qiaofu Shi, Yuan Gao, Yong Wan, Jun Zhang, Jungmok You, Yun‐Ze Long, Jie Zheng, Yusuke Yamauchi

**Affiliations:** ^1^ Shandong Key Laboratory of Medical and Health Textile Materials College of Physics Qingdao University Qingdao 266071 P. R. China; ^2^ Industrial Research Institute of Nonwovens & Technical Textiles Shandong Center for Engineered Nonwovens (SCEN) College of Textiles & Clothing Qingdao University Qingdao 266071 P. R. China; ^3^ Department of Convergent Biotechnology & Advanced Materials Science Kyung Hee University 1732 Deogyeong‐daero, Giheung‐gu, Yongin‐si Gyeonggi‐do 17104 South Korea; ^4^ School of Chemical Engineering The University of Queensland Brisbane QLD 4072 Australia; ^5^ Department of Materials Process Engineering Graduate School of Engineering Nagoya University Nagoya 464‐8603 Japan

**Keywords:** confined spaces, coordination environments, electrocatalysts, single‐atom catalysts, single‐atom nano‐islands

## Abstract

Single‐atom catalysts (SACs), renowned for their maximized atomic utilization, tunable coordination environments, and unique electronic structures, are critical to energy conversion and storage. However, obstacles to their practical performance include atomic agglomeration (caused by high surface free energy) and active site passivation (due to overly strong metal–support chemical bonds). Single‐atom nano‐islands (SANIs) catalysts, characterized by confined spaces and innovative structural designs, have better electrocatalytic activity and stability. This review comprehensively analyzes recent advancements in SANIs catalysts, highlighting their contributions to catalytic activity, stability, structural optimization, and selectivity. We systematically summarize the design principles and strategies for SANIs electrocatalysts by focusing on material selection, metal–support interactions, and coordination structures. Finally, the challenges and opportunities associated with SANIs catalysts to promote their development in heterogeneous catalysis and accelerate their transition into industrial applications are discussed.

## Graphical abstract

Single‐atom nano‐islands (SANIs) are innovative confined‐space single‐atom systems composed of single atoms, nano‐islands, and support. Due to their confined space, multiple active sites, interfacial interactions, and tunable coordination structure, SANIs catalysts exhibit excellent electrocatalytic activity, stability, and selectivity. This review comprehensively analyzes recent advancements in SANIs catalysts, and the challenges and opportunities for their development in the field of heterogeneous catalysis, and accelerating their transformation to industrial applications are discussed.

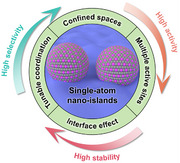

## 1 Introduction

Renewable and clean energy sources have been explored to address the threats posed by the global energy crisis and environmental pollution.^[^ ^1^ ^]^ Distinguished by its capacity to meticulously regulate the rate and selectivity of chemical reactions under an electric field, electrocatalysis exhibits remarkable promise in renewable‐energy conversion and storage.^[^ ^2^ ^]^ For electrocatalysis, catalysts are indispensable in facilitating electron transfer and optimizing the adsorption of reaction intermediates.^[^ ^3^ ^]^ Owing to their exceptional activity, favorable selectivity, and robust antioxidation properties, noble metal catalysts are widely regarded as candidates for electrocatalytic applications.^[^ ^4^ ^]^ However, the scarcity and high cost of noble metals have substantially limited their large‐scale application as electrocatalysts. It is essential to reduce the loading of noble metals while enhancing the utilization of active sites.^[^ ^5^ ^]^

**Figure 1:**
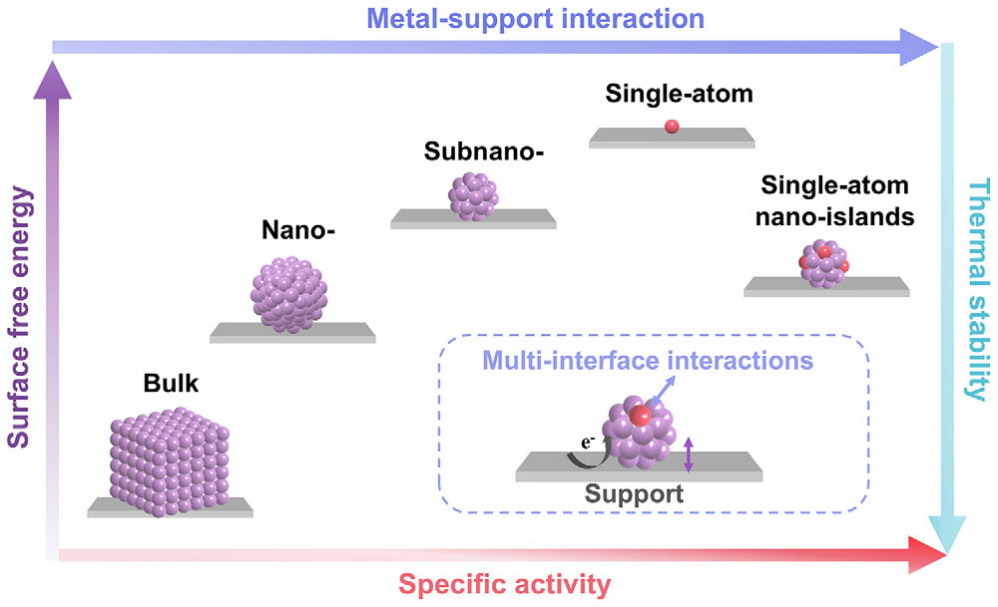
The change of surface free energy, metal–support interaction, activity, and stability of active metals with the development of catalysts.

As shown in **Figure** **1**, the development process of supported catalysts is bulk/microparticles–nanoparticles–subnanoparticles–single atoms. With the size reduction of active metal, the surface free energy and the metal–support interaction (MSI) increase. Although the catalytic activity can be increased by exposing more active sites, the size reduction destabilizes the ligands and structures of the active metal atoms, resulting in poor thermal stability. Therefore, electrocatalysts combining high activity, long‐term stability, high selectivity, and excellent conductivity are becoming increasingly crucial.^[^ ^6^ ^]^ Single‐atom catalysts (SACs) are frontrunners because of their maximized exposure to active sites, low coordination numbers, and unique atomic interface effects, endowing them with exceptional electrocatalytic performance. However, their stability remains limited in practical applications, particularly in reductive or high‐temperature environments where metal atoms are prone to aggregation, which degrades catalytic performance.^[^ ^7^ ^]^ This limitation has severely impeded the promotion of SACs from subjects of laboratory research to industrial applications.^[^ ^8^ ^]^ To address the stability issue of SACs, researchers have investigated various strategies, including the utilization of surface defects,^[^ ^9^ ^]^ enhancement of MSI,^[^ ^10^ ^]^ and use of nitrogen‐doped carbon (CN) supports,^[^ ^11^ ^]^ to optimize the coordination environment of single atoms and improve their stability. However, the stability and activity of SACs frequently exhibit a tradeoff relation. Thus, balancing high activity and long‐term stability solely by adjusting the coordination environment is challenging.^[^ ^12^ ^]^ Therefore, the advent of single‐atom nano‐islands (SANIs) catalysts marks a notable breakthrough in balancing the high activity and stability of SACs. SANIs comprise three integral parts: active‐metal atoms, nano‐islands, and support. Nano‐islands function as vital connection points, providing an optimal coordination environment for single atoms while effectively mitigating their aggregation, thereby enhancing activity and stability. In addition, there are multi‐interface interactions between the active metal atom, nano‐island and support of SANIs catalysts, which further optimizes the coordination structure and electronic structure of the active metal atoms.

Recently, SANIs have undergone substantial advancements in electrocatalysis. Its advantages over the traditional load‐type single‐atom lie in: 1) The nano‐islands in SANIs catalysts with spatial confinement can effectively inhibit the agglomeration of single atoms. 2) By designing the coordination environment of the nano‐islands, the local atomic coordination environment of the active metal atoms can be precisely regulated, which can stabilize the single‐atom sites and reduce the likelihood of structural reconstruction.^[^ ^13^ ^]^ For example, Zhang et al.^[^ ^14^ ^]^ have shown that in a specific SANIs catalyst, the strong interaction between the single metal atoms and the surrounding atoms in the nano‐island structure can significantly enhance the thermal and electrochemical stability of the single‐atom sites. In addition, Li et al.^[^ ^15^ ^]^ employed electrostatic adsorption to disperse single Pt atoms onto cerium oxide (CeO_x_) nanogels, firmly anchoring them onto a SiO_2_ support. This approach led to the high activity and remarkable long‐term stability of single Pt atoms in high‐temperature and oxygen reduction environments. Similarly, Ye et al.^[^ ^16^ ^]^ utilized CN as a support to disperse single Pt atoms onto metal oxide clusters via an electric‐pulse method. The resulting Pt_1_–MO_x_/CN catalyst demonstrated outstanding activity and stability in the oxygen reduction reaction (ORR). These findings underscore the potential of SANIs in electrocatalysis, ushering in an era of advanced catalyst design and development.

This review discusses the distinctive advantages, design criteria, and recent advancements in SANIs electrocatalysts. First, we summarize the unique merits of SANIs electrocatalysts in terms of their exceptional activity and stability, flexible structural configurations, and adaptability to diverse catalytic reaction, indicating the potential of SANIs for various electrocatalytic applications. Second, we propose design criteria and strategies for SANIs electrocatalysts based on the intricate interactions between single metal atoms, nano‐islands, and the supporting substrate in the SANIs structure. This establishes a foundation for the rational design and optimization of SANIs electrocatalysts.

Furthermore, we consolidate the recent findings on SANIs electrocatalysts in the hydrogen evolution reaction (HER), oxygen evolution reaction (OER), ORR, hydrogen oxidation reaction (HOR), and carbon dioxide reduction reaction (CO_2_RR). These reactions are vital in energy conversion and storage technologies, and the progress of SANIs electrocatalysts provides notable insights into their performance and efficiency. Finally, we delineate the future development trends and challenges associated with SANIs electrocatalysts to give a clear direction for the industrial application of SANIs and foster advancements in electrocatalysis. Overall, this review is a valuable resource for researchers and practitioners seeking to leverage SANIs electrocatalysts’ unique properties for various applications.

## 2 Advantages of SANIs Electrocatalysts

**Figure 2:**
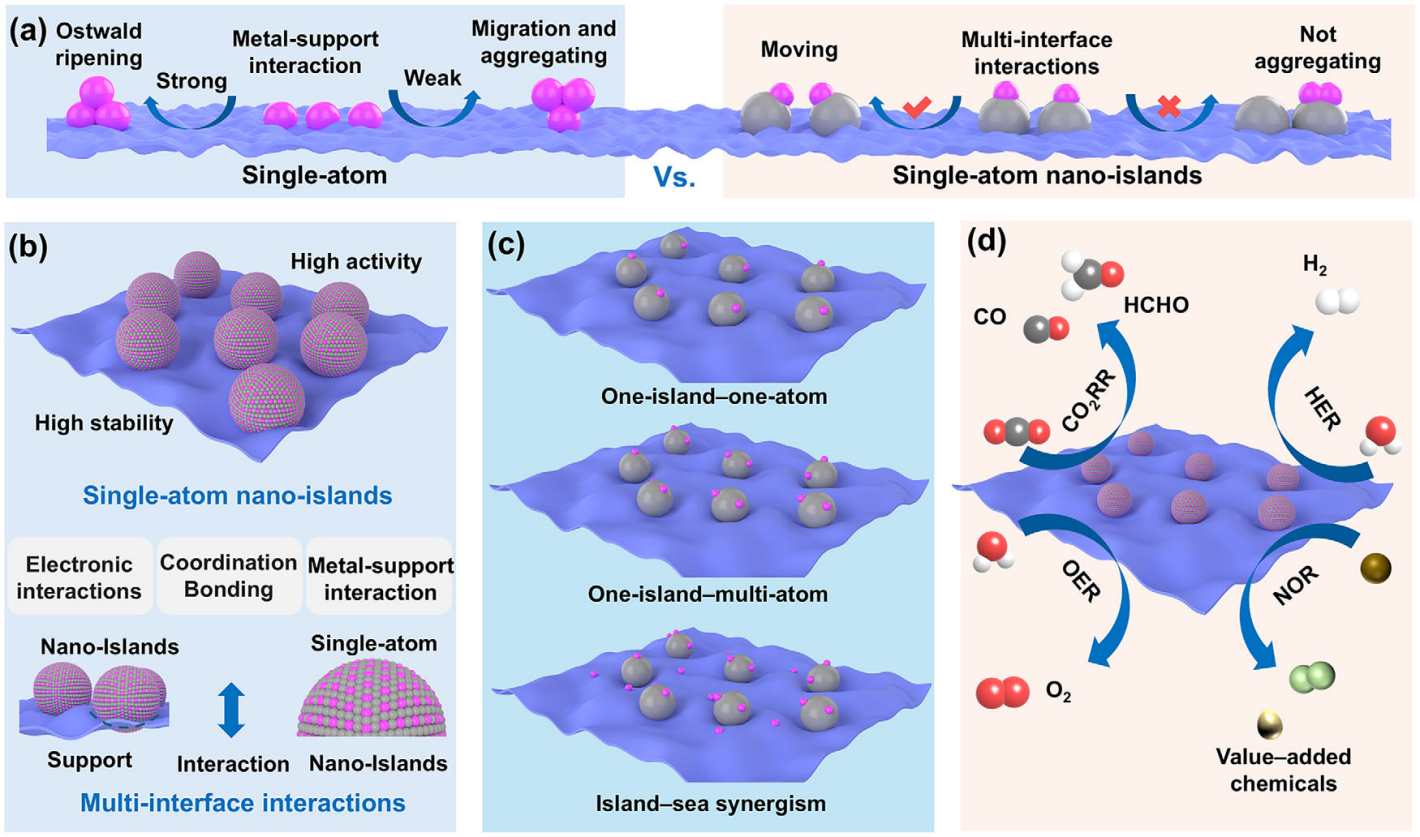
Advantages of SANIs electrocatalysts. a) Comparison of fundamental mechanisms for stability between SANIs catalysts and SACs. b) SANIs structure with both high activity and high stability dominated by multi‐interface interactions. c) Three typical types of structures of SANIs. d) Application of SANIs in different electrocatalytic reactions.

In the traditional SACs system, the agglomeration of active metal atoms on the support is the main cause of poor catalyst stability. When the MSI is too strong, the metal atoms tend to aggregate into clusters or nanoparticles via the Ostwald ripening process. However, when MSI is too weak, the metal atoms on the support tend to migrate and agglomerate (**Figure** **2**a). In contrast, the SANIs catalysts, which are innovative confined‐space single‐atom systems, comprise single active‐metal atoms, nano‐islands, and support. Due to the single active atom being confined to the nano‐island, the single atom can dynamically move within the nano‐island but not agglomerate across the nano‐island under multi‐interface interaction. In such a system, as a confined space of a single atom, the nano‐island effectively inhibits the agglomeration of metal atoms, thus ensuring the long‐term stability of the catalyst. Thus, SANIs inherit the high‐activity advantages of traditional SACs and successfully overcome the low‐stability limitation. Furthermore, owing to the flexibility in the structural composition and material diversity among single atoms, nano‐islands, and supports, SANIs catalysts are highly versatile in various electrocatalytic reactions. Herein, we elaborate on their remarkable advantages in electrocatalysis: their activity and stability, flexible structure, and adaptability to different electrocatalytic reactions.

### 2.1 With Both High Activity and Stability

Since 2011, atomically dispersed catalysts have become a major research focus across various catalytic domains owing to their exceptional atomic utilization rates and high catalytic activity.^[^ ^17^ ^]^ However, their surface free energy substantially increases when metals are dispersed at the atomic level onto supports. Consequently, metal atoms are prone to aggregation in reducing or high‐temperature environments, forming nanoclusters or nanoparticles, compromising their stability.^[^ ^12^, ^18^ ^]^ Despite developing strategies to stabilize single metal atoms, such as exploiting surface defects and MSI, balancing activity and stability is still challenging. The imbalance between activity and stability hinders the industrial application of SACs.

The incorporation of SANIs structures can address this challenge. In the SANIs structure, nano‐islands serve as conduits between the single atoms and support, offering a dispersed platform for metal atoms and effectively preventing their cross‐island migration through the confined‐space effect, thereby mitigating single‐atom aggregation in high‐temperature or reducing environments. For instance, Li et al.^[^ ^15^ ^]^ dispersed one Pt atom onto each CeO_x_ nano‐island and securely anchored the defective nano‐islands onto a SiO_2_ support with a high specific surface area. Owing to the stronger affinity of Pt atoms for CeO_x_ compared with that for SiO_2_, the Pt atoms remained active without undergoing cross‐island aggregation. The introduction of nano‐islands amplifies the interface effect and nano‐island–support interaction, augmenting the catalytic activity.^[^ ^19^ ^]^ Therefore, the multi‐interface interaction between single atoms, nano‐island and support is the key to the high activity and stability of SANIs catalysts. This multi‐interface interaction principle is consistent with the Sabatier principle of bifunctional supports proposed by Hu et al.^[^ ^20^ ^]^ The multi‐interface interactions can be classified into electron MSI, single‐atom coordination bonds, and traditional MSI (Figure 2b). Among them, there are strong electrons MSI and traditional MSI between the nano‐island and the support to prevent the migration and aggregation of the nano‐islands, thus ensuring the stability of the nano‐islands. The coordination bond and MSI between the single atom and the nano‐island ensure that the active metal is confined within the nano‐island.

Thus, the multi‐interface interactions between single atoms, nano‐islands, and support are crucial in achieving highly active and stable SANIs. Furthermore, modulating the coordination environment and electronic structure of single atoms to accelerate electron transfer and foster material transport pathways with the support is indispensable in enhancing the activity of SANIs catalysts.^[^ ^21^ ^]^ In summary, the SANIs framework introduces a paradigm for realizing SACs with concurrent high activity and stability, thereby broadening the application prospects of SACs.

### 2.2 Flexible and Adjustable Structure Model

The structural model of SANIs, comprising single metal atoms, nano‐islands, and support, provides a composition with remarkable flexibility and adjustability. In 2023, Li et al.^[^ ^12^ ^]^ introduced three prototypical SANIs structures: “one island–one atom,” “one island–multiple atom,” and “island–sea synergistic” structures (Figure 2c). These structures surmount the stability challenges associated with traditional SACs and offer prospects for excellent electrocatalytic performance. Notably, the coordination microenvironment of single atoms can be meticulously tailored by modulating nano‐islands’ electronic structure, thereby addressing the difficulty in regulating the atomic coordination environment in traditional surface‐supported single‐atom systems.

In SANIs with a “one island–one atom” structure, individual atoms migrate in the involved nano‐island without agglomerating, exhibiting robust catalyst stability. In this structural system, the interaction between active metal atoms and nano‐islands is crucial for the activity and stability of the catalysts. The MSI effect plays a key role here. Under the MSI effect, a single metal atom is paired with a metal or oxygen vacancy site on a nano‐island, thereby forming co‐localized active sites.^[^ ^22^ ^]^ During the catalytic reaction process, the single metal atom can dynamically migrate on the defect‐rich nano‐island. In this process, the coordination structure and electronic structure of the single atom may be further optimized, which is the source of the high activity of the SANIs Catalysts. For example, in defect‐rich CeO_x_ nano‐islands, a single Pt atom can migrate to different defect sites to achieve dynamic coordination, resulting in high catalytic activity.^[^ ^13^, ^15^ ^]^ In addition, the stability of the active metal atoms on the nano‐island can be precisely controlled by designing the single‐atom coordination environment (such as M‐CN, M‐O(OH)).^[^ ^16^ ^]^ In addition to the interaction between a single atom and a nano‐island, the interaction between a nano‐island and support is particularly important for the stability of the “one island–one atom” structures. The interaction is mainly driven by thermodynamics to redistribute electrons between the metal atoms and the support to achieve electronic equilibrium. The resulting atomic migration induces the formation of a stable surface structure, thereby creating a strong electron MSI between the nano‐island and the support.^[^ ^23^ ^]^ Notably, to increase the single‐atom loading of SANIs catalysts, the size of the nano‐islands is usually reduced and their density is increased. However, electron redistribution occurs under the influence of the size effect, and too strong electrons MSI may lead to nano‐islands sintering, which leads to deactivation of catalytic activity. Therefore, to improve the electrocatalytic performance, precise modulation of the density and electronic configuration of the nano‐island is imperative. Polyoxometalates (POMs), characterized by diverse structures, high modifiability, and potent electron‐ and proton‐transfer capabilities,^[^ ^24^ ^]^ are ideal nano‐island materials. Yan et al.^[^ ^25^ ^]^ leveraged the unique fourfold hollow site of POM clusters to anchor single Pt atoms; the distinctive electronic configuration induced charge dispersion in the Pt atoms, enhancing hydrogen spillover.

The “one island–multiple atom” structure exhibits heightened catalytic activity owing to multiple active single‐atom sites. The abundant active single‐atom sites on the nano‐islands may elicit electron synergy effects, bolstering the electrocatalytic performance. The strategic design of single Pt atoms with an appropriate density on nano‐islands has been proven viable.^[^ ^13^ ^]^ However, excessive single‐atom density inevitably causes agglomeration, diminishing atomic utilization and compromising catalytic activity and stability. Notably, studies have shown that the expansion of single atoms into atomic clusters or layers in the SANIs framework induces exceptional catalytic activity and stability in specific catalytic reactions.^[^ ^26^ ^]^ The formation of atomic clusters substantially increases the number of active sites and fosters metallic bonds (M–M) among multiple atoms, enhancing selectivity in complex catalytic systems.^[^ ^27^ ^]^ The confinement of single‐atom clusters in the nano‐island prevents aggregation into nanoparticles. Thus, extending SANIs to harness the synergistic effects of single atoms and single‐atom nanoclusters lays the groundwork for developing highly active, stable, and selective catalysts.

The nature of “island–sea synergistic” structure is the synergy among active metal atoms at different activity sites. This structure has multifunctional catalytic performance because it can load active metal atoms of different types and different coordination environments. On the one hand, the coordination environment of the active metal atoms on the support and the nano‐island is different, thus enabling efficient and synergistic catalysis. For example, Yang et al.^[^ ^28^ ^]^ designed SANIs catalysts for electrocatalysis of HER based on the high activity of Pt single atoms and the fast reaction kinetics of PtCo alloys. Under the synergistic effect of metal single atom and metal alloy coordination environment, the catalysts have excellent performance. Similarly, the FeMn_SA_ clusters on the nano‐islands optimize the electronic structure of the Mn atomic center, promote the adsorption of O_2_, and the cleavage of O–O bond during the ORR process. Therefore, the ORR performance was improved through the synergistic effect of highly active Mn‐N dispersed with atoms on the support.^[^ ^29^ ^]^ On the other hand, different types of active metal atoms can be loaded on nano‐islands and supports, which have unique advantages for bifunctional catalysts. For example, Xu et al.^[^ ^30^ ^]^ supported PtCo alloy nano‐islands and Co_SA_ “sea” on C_3_N_4_ support for bifunctional water splitting. Because islands and “seas” have highly active sites for OER and HER, respectively, and both of them can promote the spillover of hydroxyl or protons under a synergistic effect, so they have the maximum atomic utilization and bifunctional performance. It is worth noting that the “island–sea synergistic” structure, which has a multi‐coordinated environment of active metal atoms, has unique advantages for the small molecule electrooxidation involving multiple reaction steps and pathways. Therefore, it is possible to improve the reaction rate and achieve selective catalysis by loading active center sites of different reaction steps on “island–sea synergistic” structure.

SANIs, with both high activity and high stability, are of great significance for SACs from the laboratory to industrialization. Therefore, in the “one island–one atom” system, the characterization of the interactions between a single atom and nano‐island is crucial for guiding the synthesis and understanding the structure–activity relationship of SANIs catalysts. Typically, advanced microscopic techniques (such as aberration‐corrected transmission electron microscopy (AC‐TEM)) can characterize the interfacial structure at the atomic scale, which provides support for accurately establishing the atomic structure model of the interface between a metal atom and a nano‐island. In addition, infrared spectroscopy combined with molecular probe technology can detect the adsorption of surface‐active components of catalysts, providing a quantitative explanation for understanding the structure‐activity relationship and catalytic mechanism of SANIs. More importantly, MSI can induce electronic perturbations in active metal atoms, so the electronic structure of single atoms can be characterized by X‐ray absorption near‐edge structure (XANES). To further quantify MSI, descriptors such as surface free energy and electron density of nano‐islands can be constructed by experimental and regression methods.^[^ ^31^ ^]^ In addition, based on the concept of metal–support frontier orbital interaction. Shi et al.^[^ ^32^ ^]^ found a linear scaling correlation between catalytic activity and the lowest unoccupied molecular orbital (LUMO) position of the support, indicating that the LUMO orbital position of the support is a unified determinant of the activity and stability of SACs. The descriptor is also suitable for “one island–one atom” system, which provides a theoretical basis for the design of SANIs.

### 2.3 Adaptability to Different Electrocatalytic Reactions

Owing to their high activity and stability, SANIs catalysts are extensively employed in various electrocatalytic reactions, including the HER,^[^ ^13^, ^33^ ^]^ OER,^[^ ^11^, ^16^, ^34^ ^]^ CO_2_RR,^[^ ^35^ ^]^ ORR,^[^ ^16^, ^29^, ^36^ ^]^ and nucleophilic oxidation reaction (NOR)^[^ ^37^ ^]^ (Figure 2d).

The application of SANI catalysts in electrocatalysis offers substantial advantages. First, SANIs catalysts can maximize the utilization of metal atoms and furnish numerous active sites through unique adsorption and desorption mechanisms, enhancing the efficiency of electrocatalytic reactions. Consequently, when applied to the kinetically sluggish OER, SANIs electrocatalysts exhibit high intrinsic activity. Multiple active sites using the “island–sea synergistic” structure can be designed and implemented in water splitting, considerably reducing hydrogen production costs. Second, the robust interaction among metal atoms, nano‐islands, and the support improves the charge transfer efficiency, optimizes the reaction pathway, and enhances product selectivity. For instance, in studies on the ORR, CO_2_RR, and NOR, owing to the intricate reaction pathways and numerous reaction intermediates, catalysts must exhibit high activity while demonstrating high selectivity toward the target product.^[^ ^38^ ^]^ SANIs catalysts exhibit clear structure–activity relations; thus, theoretical simulations can help tailor the optimal configuration. For example, Ye et al.^[^ ^16^ ^]^ designed an ultrasmall FeO_x_‐based catalyst supported on ZIF‐8 to load Pt SANIs catalysts (Pt_1_–FeO_x_/CN). Compared with Pt_1_–Fe_2_O_3_/CN, the ultrasmall FeO_x_ cluster shortened the Pt–O bond distance and coordination number, achieving the optimal OH* adsorption strength for the ORR by modulating the Pt–O bond.

In summary, as an innovative confined space structure system, SANIs present a practical approach to addressing the activity and stability challenges associated with SACs. In addition, the selectivity of electrocatalytic reaction products can be improved by precisely designing the coordination environment of active metal atoms on nano‐islands. This is of great significance for multi‐path and multistep complex electrocatalytic reactions. With the discovery of novel materials, the selection of nano‐islands and supports will become increasingly diverse, broadening the application scope of SANIs catalysts in diverse catalytic reactions. However, the complexity of the structure poses challenges for the preparation of SANIs catalysts.

## 3 Rational Design of SANIs Catalyst

SANIs have exhibited exceptional activity and stability in SACs, characterized by their flexible and adjustable structures, which provide efficient catalytic systems for various reactions. The cornerstone of the SANIs design lies in the precise construction and regulation of the support, nano‐islands, and metal atomic sites. As the foundational element of SANIs structures, the support offers anchoring sites for nano‐islands and facilitates electron transport. Consequently, factors such as high specific surface area, chemical stability, interaction with nano‐islands and metal atoms, and conductivity should be considered during support selection. Currently, carbon‐based materials, including amorphous carbon, zeolite, graphdiyne (GDY), and metal–organic frameworks (MOFs), are widely employed as supports because of their outstanding performance in the abovementioned factors. Serving as bridges between the support and single metal atoms, nano‐islands provide a confined space for single metal atoms, which is critical in enhancing the stability and activity of SANIs catalysts. In the design of SANIs catalysts, the interactions among nano‐islands and single atoms, as well as their support, are critical.

Furthermore, the modulation of the electronic structure and coordination environment of nano‐islands can enhance the activity of SANIs catalysts. The material selection for nano‐islands is diverse, encompassing metal oxides, carbides, alloys, and atomic clusters, offering numerous possibilities for SANIs design. The diversity of materials and structures for support and nano‐islands necessitates the development of various methods for synthesizing SANIs. Depending on the material properties, suitable methods can be selected, such as electrostatic adsorption,^[^ ^15^ ^]^ electrophoretic deposition,^[^ ^16^ ^]^ impregnation,^[^ ^39^ ^]^ hydrothermal synthesis,^[^ ^13^ ^]^ and self‐assembly.^[^ ^40^ ^]^ The flexible application of such methods provides robust support for precise SANIs construction. Several key scientific challenges exist in designing metal atom sites for SANIs catalysts: 1) How to increase the loading of metal atoms while suppressing aggregation, 2) how to regulate the local coordination structure of single‐atom sites to enhance catalyst performance, 3) how to design single‐atom synergistic catalysts to achieve synergistic catalysis among multiple single atoms, and 4) how to develop macroscopic preparation methods for SANIs catalysts, thereby paving the way for their industrial applications. These challenges must be addressed to promote SANIs catalysts.

### 3.1 SANIs Structure Substrate—Support

The design of the support influences the SANIs catalysts’ activity and stability. Specific SANIs structures can be designed by controlling the specific surface area of the support and the loading methodology of the nano‐islands. An increase in surface‐supported nano‐islands can be achieved by selecting materials with a large specific surface area. Conversely, internally supported nano‐islands enhance the interaction between active sites through unique structures, such as core–shell architectures and hollow carbon nanotubes, thereby improving the corrosion resistance of the catalyst. The choice of support materials impacts the loading and catalytic performance of nano‐islands. The support's highly conductive and porous structure accelerates electron transfer, facilitating material transport.

**Figure 3:**
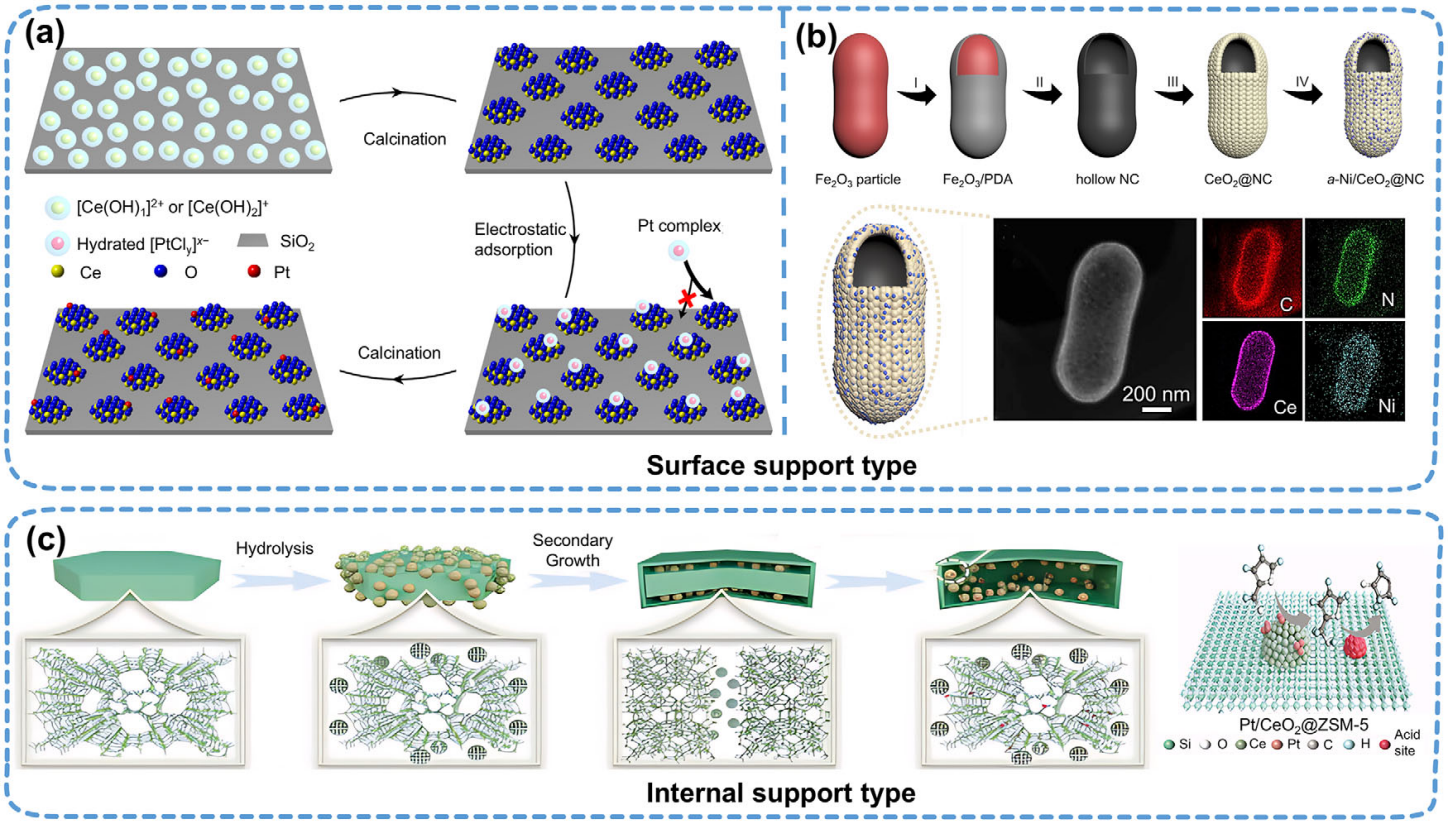
Influence of the support on the design of SANIs catalysts. a) Electrostatically adsorbed CeO_x_ nano‐islands were directionally anchored to the SiO_2_ support to synthesize SANIs. Reproduced (Adapted) with permission.^[^ ^15^ ^]^ Copyright 2022, Springer Nature. b) Schematic of Ni/CeO_2_@NC SANIs preparation on a high specific surface area NC support surface and its high‐angle annular dark‐field scanning transmission electron microscopy and energy‐dispersive X‐ray spectroscopy elemental maps. Reproduced (Adapted) with permission.^[^ ^11b^ ^]^ Copyright 2023, American Association for the Advancement of Science. c) Synthesis of encapsulated‐structure Pt/CeO_2_@ZSM‐5 SANIs through the “confined autoredox” strategy. Reproduced (Adapted) with permission.^[^ ^42^ ^]^ Copyright 2024, Wiley‐VCH.

Currently, nano‐islands are predominantly anchored to the support through surface‐supported methods. SANIs can be designed by precisely controlling the electrostatic adsorption between the support and nano‐islands. Li et al.^[^ ^15^ ^]^ harnessed the electronegativity of a SiO_2_ support in alkaline solutions to achieve the selective loading of CeO_x_ nano‐islands via electrostatic adsorption (**Figure** **3**a). With its large specific surface area, the NC support is advantageous for synthesizing SANIs catalysts. Pei et al.^[^ ^11b^ ^]^ designed a peanut‐shaped hollow NC support and synthesized a Ni/CeO_2_@NC SANIs catalyst by dispersing single Ni atoms onto CeO_2_ nano‐islands (Figure 3b). During OER tests, Ni/CeO_2_@NC exhibited an overpotential of only 286 mV at a current density of 10 mA cm^−2^. However, Ni/CeO_2_ without the NC support underwent agglomeration. Thus, the high stability of SANIs systems is attributable to the confining effect of nano‐islands on single atoms and to the crucial role of the support in inhibiting single‐atom aggregation.

The corrosion resistance and durability of SACs are paramount in industrial applications.^[^ ^41^ ^]^ The internally supported structure has notable advantages in mitigating the corrosion of acidic electrolytes at the active site. Zhao et al.^[^ ^42^ ^]^ prepared a Pt/CeO_2_@ZSM‐5 SANIs catalyst with high activity and selectivity using encapsulated CeO_2_ nano‐islands loaded with single Pt atoms on the inner wall of ZSM‐5 zeolite through a “confined autoredox” strategy (Figure 3c). This encapsulation structure enhances the interaction between active single‐Pt‐atom sites and acids and promotes the orderly migration of reactants and intermediates at different active sites.

Zeolite materials exhibit unique advantages in designing SANIs catalysts, attributable to their microporous structure and tunable acidity. Encapsulating metal active sites in the zeolite framework results in an effective confined‐space effect, furnishing Brønsted and Lewis acid sites.^[^ ^43^ ^]^ Encapsulating precious metals in acidic zeolites can improve catalytic activity and product selectivity.^[^ ^43^, ^44^ ^]^ Furthermore, the proximity of metal–acid active sites influences reaction activity and product selectivity in catalytic reactions involving multiple pathways.^[^ ^45^ ^]^ Consequently, this encapsulation strategy enhances catalytic performance and selectivity while providing an approach to modulating metal–acid interactions. GDY provides an excellent environment for the uniform dispersion and stable growth of nano‐islands owing to its uniformly distributed macroporous structure, high conductivity, and porous architecture.^[^ ^37^ ^]^ GDY's uneven charge distribution and conjugated alkyne‐rich characteristics endow it with potent electron acceptor or donor capabilities, sustaining a high catalytic activity and structural stability over long periods.^[^ ^46^ ^]^ Beyond conventional 1D and 2D materials, 3D hollow carbon nanocages have demonstrated considerable potential in designing SANIs catalysts because of their adjustable structural morphology, multilevel pore channels, and high conductivity.^[^ ^47^ ^]^ By introducing appropriate nano‐islands and metal atoms onto these nanocages, efficient SANIs catalysts can be formulated for diverse catalytic reactions.

### 3.2 SANIs Structural Connection Points—Nano‐Islands

As platforms for loading single metal atoms, nano‐islands serve dual functions: they restrict the migration and aggregation of single atoms and enable precise control over the coordination microenvironments of the atoms through rational design. Therefore, the selection of nano‐islands is crucial for the synthesis and performance of SANIs catalysts. Generally, the selection criteria of nano‐islands include three aspects: 1) The MSI between the nano‐islands and the support should be strong enough to ensure the stability of the nano‐islands during the electrocatalytic reaction; 2) The affinity of the nano‐islands to the active metal atoms should be much larger than that of the support, so as to ensure that the nano‐islands can capture enough active metal atoms; 3) The strong electronic and coordination interactions between nano‐islands and active metal atoms enhance the electrocatalytic performance. It is noteworthy that in order to improve the overall catalytic activity of the SANIs catalysts with “one island–one atom” structure, the size of the nano‐islands is usually reduced and their density is increased. However, under severe reaction conditions (temperature, reaction atmosphere, current density, pH, etc.), too strong MSI will be generated between the ultrasmall nano‐islands and the support, resulting in the Ostwald ripening of the nano‐islands and the inactivation of the active atomic sites. Therefore, it is an unreasonable strategy to prepare nano‐islands with ultrasmall size and high density under severe reaction conditions. In the SANIs catalysts synthesis strategies, the precise capture and stabilization of active metal atoms by designing atomic coordination, electrostatic adsorption, and single‐atom alloy strategies of nano‐islands are worthy of attention.

**Figure 4:**
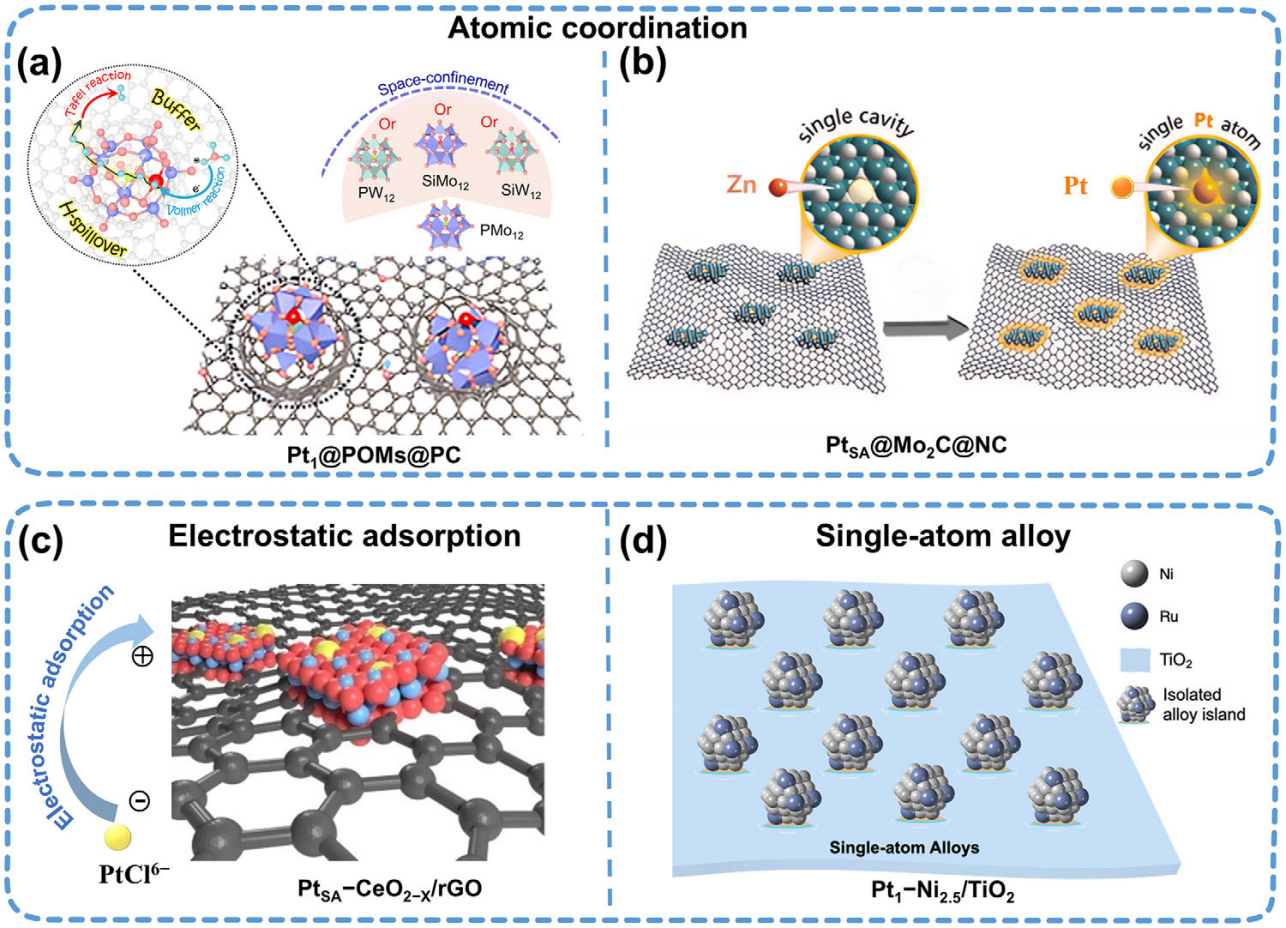
Fine design and regulation of nano‐islands in a SANIs catalyst structure. a,b) Atomic coordination capture strategy to synthesize SANIs. Reproduced (Adapted) with permission.^[^ ^21^, ^25^ ^]^ Copyright 2024, The Royal Society of Chemistry. Syntheses of SANIs via c) an electrostatic‐adsorption strategy, Reproduced (Adapted) with permission.^[^ ^13^ ^]^ and d) a single‐atom alloy strategy. Reproduced (Adapted) with permission.^[^ ^54^ ^]^ Copyright 2024, Wiley‐VCH.

The formation of stable coordination structures via atomic coordination is effective for trapping and stabilizing active metal atoms. For instance, robust coordination structures originate between metal cations and elements such as N, O, and S, which possess lone pair electrons.^[^ ^48^ ^]^ Given the diversity of oxygen species, POMs can capture Pt and other precious‐metal atoms via M–O coordination, forming stable coordination structures, such as Pt–O bonds. Consequently, the migration of single atoms is constrained, enhancing their catalytic activity (**Figure** **4**a). SANIs’ catalytic performance can be further optimized by precisely modulating the coordination structure of M–O.^[^ ^39^ ^]^ In addition, atomic defects are a powerful means to trap active metal atoms, enabling the synthesis of SANIs through the “defect substitution” of atoms in nano‐islands. Yue et al.^[^ ^21a^ ^]^ introduced a hierarchical stabilization system. They designed a highly active and stable Pt_SA_@Mo_2_C@NC SANIs catalyst by substituting local Zn defects with single Pt atoms (Figure 4b). In this catalyst, the single Pt atoms are constrained by the defect sites of the Mo_2_C lattice and form a robust electronic interaction with the axial N coordination on the substrate, effectively stabilizing the single Pt atoms and enhancing the overall structural stability.

Single‐atom‐oriented electrostatic adsorption to nano‐islands can be achieved using the charge difference between different groups. Liu et al.^[^ ^13^ ^]^ employed a one‐step hydrothermal method to disperse Pt_SA_ on positively charged CeO_2−x_ via electrostatic adsorption (Figure 4c). Pt_SA_ with different densities was obtained by adjusting the distribution density of the CeO_2−x_ nano‐islands on reduced graphene oxide (rGO). Among them, the middle density of the SANIs exhibited the best HER activity. The size of nano‐islands is a key factor affecting the catalytic performance of SANIs. The distribution density and coordination number of single atoms can be optimized by accurately adjusting the nano‐island size. Thereafter, the catalytic activity can be controlled. For example, Muravev et al.^[^ ^49^ ^]^ synthesized atomically dispersed Pd–CeO_2_ catalysts with variable CeO_2_ nano‐islands with 4–18 nm sizes for CO oxidation via flame spray pyrolysis. The smaller Pd–CeO_2_ catalysts exhibited strong MSIs and exceptional CO oxidation activity.

Noteworthily, single‐atom alloys (SAAs), with their unique nano‐island structures, exhibit distinctive catalytic properties during electrocatalytic reactions. Dissimilar to conventional metal alloys, the active metal sites in SAAs are atomically dispersed in the host metal.^[^ ^50^ ^]^ Given the multiple dispersed single metal atoms on SAAs, they can function as nano‐islands to form “island–sea synergistic” structures with other metal atoms or “one island–multiple atom” structures for electrocatalytic reactions. Current synthesis methods for SAAs include Co reduction,^[^ ^51^ ^]^ atomic layer deposition,^[^ ^52^ ^]^ and sequential reduction.^[^ ^53^ ^]^ However, synthesizing SAAs with ultrasmall particles using these methods is challenging. Zou et al.^[^ ^54^ ^]^ continuously refined alloy particles to produce an RuNi SAA catalyst with a size of ≈1 nm to obtain nanoscale SAAs (Figure 4d). In this catalyst, Ru is atomically dispersed in the host Ni metal, forming a special electronic structure that results in high CO selectivity.

In summary, nano‐islands are crucial in designing SANIs catalysts. By precisely modulating the materials, sizes, and interactions of nano‐islands, the catalytic performance of SANIs can be optimized and enhanced. Future research should continue to explore novel nano‐island materials, more precise size‐regulation techniques, and more efficient single‐atom‐capture strategies to promote the application of SANIs catalysts. In preparing SANIs electrocatalysts, the isolation and ionic characteristics of the supported single atom are critical for its activity and selectivity. However, SANIs electrocatalysts may not be feasible for electrocatalytic reactions requiring two or more adjacent active‐metal atoms. Thus, the accurate construction of multiatom nano‐island electrocatalysts based on SANIs is a notable future development direction, offering possibilities for a more efficient and stable electrocatalytic performance. Notably, single‐atom cluster nano‐island catalysts have garnered substantial attention because of their abundant active sites. Common synthesis methods include atomic layer deposition,^[^ ^55^ ^]^ template‐assisted synthesis,^[^ ^56^ ^]^ and electrochemical reduction.^[^ ^57^ ^]^ The ongoing development of these methods provides additional options and possibilities for the design and application of SANIs catalysts. In addition to fully exploiting the limiting activity and selectivity of SANIs electrocatalysts, theoretical calculations and machine learning are used to guide the rational design and precise synthesis of SANIs catalysts, which can provide a theoretical basis for the design and synthesis of nano‐island electrocatalysts. This guides the regulation of the micro‐coordination environment of the active metal atoms on the nano‐islands.

## 4 Application Progress of SANIs in Electrocatalysis

In electrocatalysis, noble metals are esteemed for their exceptional catalytic properties. However, their high cost and scarcity impede large‐scale applications. Consequently, achieving maximum catalytic efficiency with minimal dosage to mitigate costs has become a major research focus. Although SACs can maximize the exposure of active sites, they are prone to agglomeration and structural reconstruction during electrochemical reactions, thereby reducing the stability of the catalysts.^[^ ^5^, ^58^ ^]^ The recent advent of SANIs catalysts presents a solution to this challenge. Its advantage lies in that the nano‐islands with spatial confinement inhibit the agglomeration of single atoms, and the atomic coordination environment of active metal atoms on the nano‐islands can be precisely designed. Therefore, the development of SANIs has provided strong support for SACs from laboratory research to industrial applications.

Furthermore, the versatility in selecting single metal atoms, nano‐islands, and support materials, along with interactions among these components, enables SANIs catalysts to adapt to diverse electrocatalytic reactions. Incorporating spatially confined nano‐islands mitigates the aggregation of single atoms, resulting in high activity and stability in electrocatalysis. This section discusses the application of SANIs electrocatalysts in the HER, the OER, fuel cells, zinc–air batteries, the CO_2_RR, and the NOR.

### 4.1 SANIs Catalysts in HER and OER

Electrolytic water splitting—an efficient and ecofriendly hydrogen production method—has garnered considerable attention. However, current membrane electrode assembly (MEA) electrolyzers still rely on precious‐metal catalysts as electrodes. The sluggish kinetics of the anodic OER substantially limit hydrogen production efficiency.^[^ ^59^ ^]^ Optimal catalysts for water electrolysis should have the appropriate adsorption strength for intermediates (such as H* in the HER as well as O*, HO*, and HOO* in the OER) and long‐term stability.^[^ ^60^ ^]^ SANIs electrocatalysts’ structural diversity facilitates the modulation of the electronic structure of the catalyst surface, thereby enhancing catalytic activity. In addition, the high stability of atomically dispersed precious metals in the SANIs structure ensures applicability in acidic and alkaline environments, fostering the development of industrial MEA electrolyzers.

**Figure 5:**
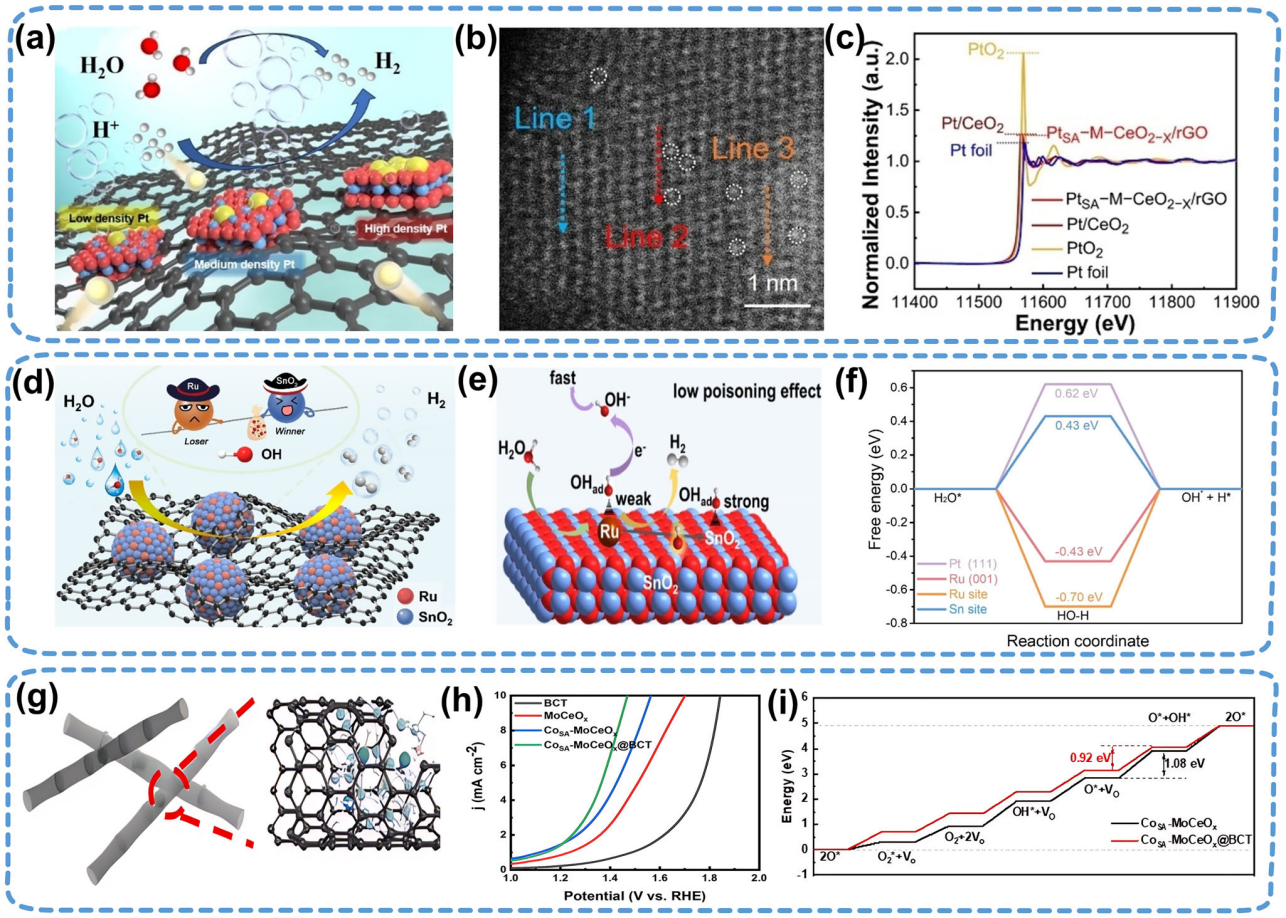
Application of SANIs catalysts in the HER and OER. a) Schematic of a Pt_SA_–M–CeO_2−x_/rGO SANIs catalyst for electrolytic water splitting in an HER. b) High‐angle annular dark‐field scanning transmission electron microscopy image of the Pt_SA_–M–CeO_2−x_/rGO SANIs electrocatalyst. c) X‐ray absorption near edge structure spectra of Pt in the Pt_SA_–M–CeO_2−x_/rGO electrocatalyst. Reproduced (Adapted) with permission.^[^ ^13^ ^]^ Copyright 2024, Wiley‐VCH. d) Schematic of the Ru_SA_–SnO_2_/C electrocatalyst for water splitting in the HER. e) Schematic of the HER activity enhancement mechanism of the Ru_SA_–SnO_2_/C catalyst. f) Free energy of hydrolysis of the Ru_SA_–SnO_2_/C catalyst at different sites. Reproduced (Adapted) with permission.^[^ ^33d^ ^]^ Copyright 2022, Wiley‐VCH. g) Schematic of the Co_SA_–MoCeO_x_@BCT catalyst structure. h) Polarization curve of the Co_SA_–MoCeO_x_@BCT catalyst. i) Free energy barrier of each step in the Co_SA_–MoCeO_x_@BCT catalyst–based OER. Reproduced (Adapted) with permission.^[^ ^34^ ^]^ Copyright 2024, Elsevier.

Pt is the benchmark electrocatalyst for the HER because of its optimal hydrogen adsorption capacity. The development of single‐atom Pt catalysts can improve atomic utilization; however, their high surface energy tends to promote the aggregation of Pt particles, thereby limiting practical applications. Thus, advancing Pt‐based SANI HER catalysts with confined‐space structures is crucial in promoting large‐scale hydrogen production in electrolyzers. For instance, Pt_SA_–M–CeO_2−x_/rGO SANIs catalysts have been synthesized by dispersing Pt_SA_ with varying densities on CeO_2−x_ nano‐islands with oxygen vacancies, with graphene as the support (**Figure** **5**a).^[^ ^13^ ^]^ A high‐angle annular dark‐field scanning transmission electron microscopy (HAADF‐STEM) image (Figure 5b) and X‐ray absorption near edge structure spectra (Figure 5c) confirmed the uniform and directional dispersion of Pt as single atoms on CeO_2−x_ nano‐islands. The catalyst exhibited exceptional HER performance in all pH environments and maintained stable operation for 90 h in a 0.5 m H_2_SO_4_ electrolyte at a current density of −150 mA cm^−2^. DFT calculations revealed that introducing CeO_2−x_ nano‐islands induced variations in the atomic distance, optimizing the H* intermediate adsorption, and enhancing the HER performance.

Moreover, the robust chemical bond formed by alloying Pt_SA_ with transition metals considerably improves the involved material's resistance to acid corrosion, with exceptional HER activity and stability over a broad pH range.^[^ ^61^ ^]^ Yang et al.^[^ ^28^ ^]^ synthesized a Pt_SA_ SANIs catalyst with the “island–sea synergistic” structure using Pt–Co alloy as the nano‐islands and ZIF‐67 as the support, confirming its excellent HER properties under alkaline and acidic conditions. Owing to the synergistic effect between the Co–Pt alloy and Pt_SA_, the catalyst exhibited a high *d*‐band occupancy rate, providing numerous free electrons to facilitate H* conversion and resulting in fast HER kinetics. Ru is a promising catalyst owing to its Pt‐like electronic structure and lower cost than Pt. However, Ru's strong binding to the adsorbed hydroxyl (OH_ad_) results in active site poisoning, limiting the HER performance.^[^ ^33b^ ^]^ In the structural design of SANIs, the coordination microenvironment of Ru_SA_ can be tailored through the judicious selection and regulation of nano‐islands to inhibit Ru site poisoning. For instance, introducing oxyphilic species (such as WC_x_ or SnO_2_) to enhance the strong binding effect of OH_ad_ and perform competitive adsorption with Ru can effectively alleviate the OH_ad_ poisoning effect at the Ru active site.^[^ ^33^, ^62^ ^]^ Zhang et al.^[^ ^33d^ ^]^ synthesized an SANIs catalyst with Ru_SA_ anchored on SnO_2_ nano‐islands on a carbon support via competitive adsorption (Figure 5d). DFT calculations revealed that the competitive adsorption of OH_ad_ by SnO_2_ effectively inhibited OH_ad_ poisoning at the Ru active site (Figure 5e,f).

For the OER, the sluggish kinetics and high thermodynamic energy barriers have necessitated a focus on catalysts that ensure high atomic utilization of noble metals, such as Ir and Ru. However, in acidic environments, the interaction between single atoms and their supports tends to weaken, destabilizing the atomic coordination structure. Consequently, lattice oxygen can readily disrupt the coordination of single atoms during the OER, leading to corrosion or dissolution. Kumar et al.^[^ ^63^ ^]^ addressed this challenge with remarkable OER activity and durability for single Ir atoms under acidic conditions by precisely modulating the coordination environment of Co_3_O_4_ nano‐islands. Furthermore, the internally supported SANIs catalyst effectively mitigates the corrosion of the active sites by an acidic electrolyte. Liu et al.^[^ ^34^ ^]^ introduced a strategy to enhance catalyst corrosion resistance using bamboo‐like carbon nanotubes coated with MoCeO_x_‐supported single Co atoms (Figure 5g). Their experimental results show that Co_SA_‐MoCeO_x_@BCT remains stable for >60 h in a proton‐exchange membrane electrolyzer coated with carbon nanotubes, exhibiting an overpotential of only 239 mV at a current density of 10 mA cm^−2^ in 0.5‐m H_2_SO_4_ (Figure 5h). In situ Raman spectroscopy and DFT calculations reveal that incorporating MoCeO_x_ nano‐islands facilitates the transformation of single‐Co‐atom sites into highly active Co^3+^–O sites with a low coordination number and abundant oxygen vacancies. This enhances the activity of lattice oxygen during the OER, reduces the energy barrier of the rate‐determining step in the lattice oxygen‐mediated pathway, and elevates the OER activity in acidic environments (Figure 5i).

Despite their high mass activity and stability in HER and OER applications, SANIs catalysts have seldom achieved industrial current densities (>500 mA cm^−2^). Thus, the enhancement of SANIs electrocatalysts’ current density and long‐term stability will be explored. By optimizing the catalytic structure and composition and investigating preparation methodologies and support materials, the large‐scale application of SANIs catalysts in hydrogen production via electrolytic water splitting and fuel cells can be advanced. Moreover, beyond freshwater electrolysis, exploring SANIs electrocatalysts for seawater electrolysis for hydrogen production merits considerable attention.^[^ ^64^ ^]^ Through the selection of nano‐island materials and surface modification design, SANIs electrocatalysts have the potential to have excellent corrosion resistance in seawater electrolysis for hydrogen production.

### 4.2 SANIs Catalyst is Applied to Fuel Cells and Zinc–Air Cells

**Figure 6:**
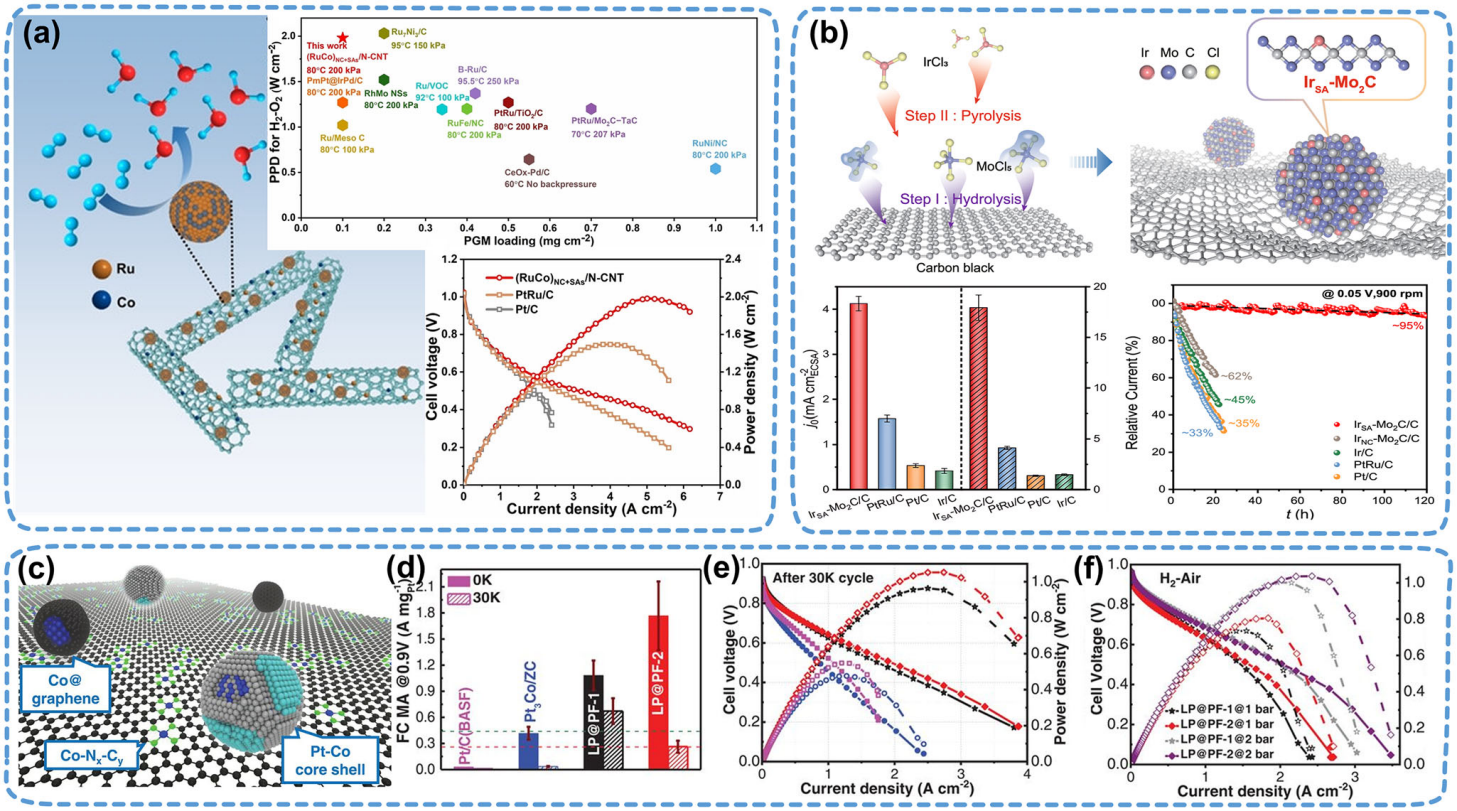
Application of SANIs catalysts in fuel and zinc–air cells. a) (RuCo)_NC+SAs_/N–CNT SANIs electrocatalyst used in the HOR. Reproduced (Adapted) with permission.^[^ ^69^ ^]^ Copyright 2024, Wiley‐VCH. b) The Ir_SA_–Mo_2_C/C SANIs electrocatalyst exhibits high activity and stability when used in the HOR. Reproduced (Adapted) with permission.^[^ ^70^ ^]^ Copyright 2024, Springer Nature. c) Schematic of an ultralow‐Pt‐content SANIs catalyst (LP@PF) with Pt–Co core–shell nanoparticles and Co–N_x_–C_y_. d) The catalytic activity of the LP@PF catalyst before and after the 30‐K cycle at 0.9 V. e) I–V polarization and power density of the LP@PF catalyst after an H_2_–O_2_ fuel cell stability test. f) Performance of the LP@PF catalyst when used in H_2_–air fuel cells. Reproduced (Adapted)with permission.^[^ ^36a^ ^]^ Copyright 2018, American Association for the Advancement of Science.

As the cornerstone of current and future hydrogen energy utilization, fuel cells spearhead new‐energy transformation exploits. Among various fuel cell systems, hydroxide‐exchange membrane fuel cells (HEMFCs) hold extensive application potential because of their reduced reliance on Pt‐group precious metals and potential cost savings.^[^ ^65^ ^]^ However, the sluggish kinetics of the OER primarily rely on high‐load Pt‐group catalysts, hindering further enhancement in performance.^[^ ^26^, ^66^ ^]^ Similarly, the ORR occurring at the cathode in fuel cells and zinc–air batteries urgently necessitates low‐load noble metal or even non‐noble metal catalysts. In the discharge process of zinc–air batteries, the reactivity of the cathodic ORR is the major factor limiting its performance.^[^ ^67^ ^]^ SANIs electrocatalysts, characterized by their atomically dispersed active sites, single‐atom stability, and the synergistic effect among the nano‐islands, support, and single atoms, offer a strategy for modulating reaction intermediates and achieving efficient HOR and ORR in diverse environments. The adsorption strength of *H governs the catalytic activity of HOR and *OH intermediates.^[^ ^68^ ^]^ Although Pt‐group metals are the most promising HOR catalysts, developing HOR catalysts that are cost‐effective, highly active, and resistant to CO poisoning remains challenging. Cui et al.^[^ ^69^ ^]^ synthesized (RuCo)_NC+SAs_/N–CNT SANIs electrocatalysts with remarkable HOR activity (1.98 W cm^−2^ at a loading of 0.1 mg_Ru_ cm^−2^), anti‐CO‐poisoning properties, and long‐term durability, achieved through Ru/Co atom dispersion and alloying modifications (**Figure** **6**a). This indicates that the synergistic interaction between RuCo dilute alloy nanoparticles and single Ru/Co atoms optimizes the adsorption of *H, *OH, and *CO on the catalyst surface, thereby exhibiting exceptional HOR properties even at low noble‐metal loadings. The choice of nano‐island materials is crucial to further decrease the precious‐metal loading while maintaining high HOR activity. With its Pt‐like electronic structure, Mo_2_C is an ideal nano‐island candidate. As illustrated in Figure 6b, the Ir_SA_–Mo_2_C/C SANIs electrocatalyst has been prepared via strong interaction between Mo_2_C and Ir atoms and applied in an HOR, achieving the homogeneous dispersion and stability of single Ir atoms.^[^ ^70^ ^]^ The catalyst exhibits exceptionally high exchange current density (4.1 mA cm^−2^_ECSA_), mass activity (17.9 A mg_Ir_^−1^ at 0.05 V vs reversible hydrogen electrode), and stability (120 h). The peak power of an HEMFC assembled at a low Ir loading (0.05 mg_Ir_ cm^−2^) reaches 1.64 W cm^−2^. DFT calculations revealed that introducing Mo_2_C nano‐islands optimized the Gibbs hydrogen adsorption free energy (ΔGH*) of single Ir atoms and enhanced OH adsorption, achieving the fastest reaction kinetics. The interfacial water structure is another crucial factor influencing the HOR rate. Among the three SANIs structures, the “island–sea synergistic” structure is ideal for the HOR owing to its unique electron transfer/material transport channels and component interactions. Notably, metal alloy particles (such as alloys formed by Ir and transition metals, Ir and Ru, and Pt and Ru) exhibit exceptional HOR activity when functioning as nano‐islands.^[^ ^71^ ^]^

The sluggish kinetics of the cathodic ORR and instability observed under high‐activity conditions impede the widespread application of fuel cells. Integrating Pt‐based nanoparticles with M–N–C (metal–nitrogen–carbon) structures can enhance catalytic stability and activity.^[^ ^72^ ^]^ Chong et al.^[^ ^36a^ ^]^ successfully synthesized an ultralow‐Pt‐content catalyst comprising Pt–Co core–shell nanoparticles and Co–N_x_–C_y_ using an MOF as the support (Figure 6c). The catalyst exhibited exceptionally high mass‐specific activity (1.77 A mg_Pt_^−1^) during an ORR test (Figure 6d). Furthermore, it demonstrated robust catalytic activity and stability when used in H_2_–air and H_2_–O_2_ fuel cells (Figure 6e,f). DFT calculations revealed the interplay between the Pt–Co alloy and Co–N_x_–C_y_, which facilitated the transfer and reduction of H_2_O_2_ as well as mitigated the corrosion of the support and single‐atom sites. SANIs electrocatalysts have shown considerable promise in ultralow‐temperature zinc–air batteries. Leveraging the highly conductive ZIF‐8 as support, Ye et al.^[^ ^16^ ^]^ synthesized a Pt_1_–FeO_x_/CN catalyst with a “one island–one atom” structure anchored by FeO_x_ clusters to atomically dispersed Pt via an electric‐pulse method. The catalyst exhibited outstanding ORR activity in an alkaline environment and maintained peak power density (45.1 mW cm^−2^) and long‐term stability in zinc–air batteries at an ultralow temperature of −40 °C. Theoretical calculations revealed that FeO_x_ clusters shorten the Pt–O bond distance, achieving optimal OH* adsorption strength and inducing exceptional ORR activity even under harsh conditions. In summary, whether in the HOR or ORR, SANIs electrocatalysts can adapt to diverse reaction environments and complex reaction intermediates because of their flexible characteristics, offering insights into improving the performance of fuel cells and zinc–air batteries.

Although SANIs catalysts can achieve excellent HOR and ORR performance by adjusting the local coordination environment and electronic structure of the active metal atoms on the nano‐islands. However, the single active metal site is difficult to efficiently achieve the adsorption of oxygen‐containing intermediates (e.g. *OOH, *O, and *OH) and bond cleavage in the four‐electron ORR process.^[^ ^73^ ^]^ Therefore, the construction of “island–sea synergistic” and diatomic‐ or multi‐atom systems of SANIs electrocatalysts to enhance the synergistic effect of different active metals provides an encouraging strategy for improving the reaction rate and selectivity of ORR.

### 4.3 SANIs Catalyst Applied in CO_2_RR and CORR

Electrocatalytic CO_2_RR/CORR conversion is one of the most appealing avenues for global carbon neutrality. In particular, CO_2_/CO can be transformed into high‐value reduction products using electrocatalysts, encompassing C1 products (such as methanol, formic acid, and methane), C2 products (e.g., ethanol, acetic acid, and ethylene), and higher carbon chain alcohols and alkanes.^[^ ^74^ ^]^ The coupling of C−C bonds during the CO_2_RR/CORR is important as highly reduced C2 products possess a high economic value in industrial production. However, the reaction pathway's complexity and the intermediates’ diversity pose notable challenges in selectively reducing CO_2_/CO to high‐value C2 products.

**Figure 7:**
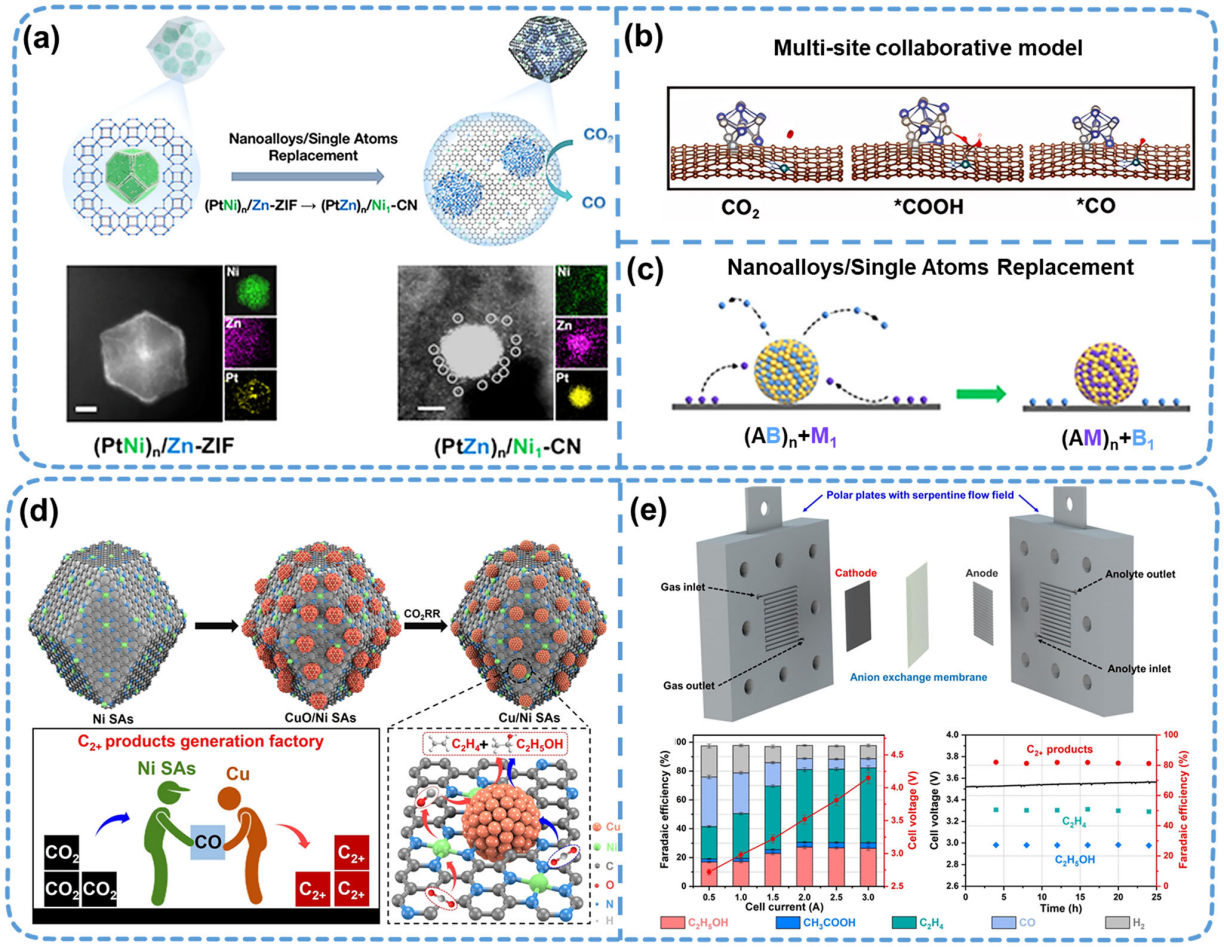
Application of SANIs catalysts in the CO_2_RR and CO reduction reaction. a) Synthesis of a (PtZn)_n_/Ni_1_–CN catalyst and energy‐dispersive spectroscopy elemental maps. b) Synergistic model of the (PtZn)_n_/Ni_1_–CN catalyst active site in the CO_2_RR. c) Atomic displacement between single atoms and the nanoalloy. Reproduced (Adapted) with permission.^[^ ^35^ ^]^ Copyright 2022, American Chemical Society. d) Schematic of the synthesis and reaction mechanism of a CuO/single‐Ni‐atom tandem catalyst. e) CuO/single‐Ni‐atom tandem catalyst for the CO_2_RR in an MEA electrolyzer. Reproduced (Adapted) with permission.^[^ ^79^ ^]^ Copyright 2018, Springer Nature.

SACs exhibit exceptional activity and selectivity in the CO_2_RR/CORR owing to their unique atomic structure and electronic properties. For instance, high selectivity toward C2 products is achievable by designing single‐atom coordination structures that favor C−C coupling.^[^ ^75^ ^]^ However, the practical deployment of SACs is severely constrained by their low active site loading and poor stability.^[^ ^76^ ^]^ Spatially confined SANIs hold the potential to realize the CO_2_RR/CORR with high activity, selectivity, and stability. The design of metal alloy nano‐islands or single atomic clusters by leveraging multi‐active‐site synergies is crucial for the high activity and selectivity of the CO_2_RR/CORR. Furthermore, the interaction between single‐atom sites, nano‐islands, and the support in the “island–sea synergistic” structure system is vital in achieving high activity and selectivity. Wang et al.^[^ ^35^ ^]^ successfully constructed an “island–sea synergistic” structure through an atomic replacement approach, enabling the displacement of metal atoms in heterogeneous catalysts. In an inert gas atmosphere at high temperatures, the Ni atom in Pt–Ni alloy nano‐islands could be replaced by the Zn atom in the CN support, thereby forming a (PtZn)_n_/Ni_1_–CN catalyst (**Figure** **7**a). DFT calculations indicated that the introduced Pt–Zn alloy could adsorb and stabilize the *COOH intermediate through Pt–O bond interaction, facilitating *CO desorption and exhibiting superior CO_2_RR performance (Figure 7b). This work presents a mode for realizing metal element displacement in heterogeneous catalysts (Figure 7c). It achieves the interconversion of single metal atoms between nanoalloys, providing a fresh perspective for developing efficient and highly selective CO_2_RR catalysts.

In the catalytic system of SANIs, agglomeration between atoms may decrease activity when atomic spacing is reduced or the load is excessively high. However, in complex reaction systems, M–M originating from atomic agglomeration occasionally facilitates the cleavage and formation of reactant chemical bonds and the activation and adsorption/desorption of intermediates.^[^ ^77^ ^]^ Zhang et al.^[^ ^78^ ^]^ introduced a “single‐atom‐layer cluster” structure, which substantially enhances reaction selectivity by constructing high‐density single atoms with ultrasmall atomic spacing in various regions on the support. Furthermore, the synergistic effect between single atoms and ultrasmall nanoparticles can improve the activity and selectivity of the CO_2_RR. Zhang et al.^[^ ^79^ ^]^ designed a CuO/single‐Ni‐atom tandem catalyst by leveraging the synergistic interaction between single Ni atoms and CuO nanoparticles on ZIF8 to achieve precise control over the CO_2_RR pathway (Figure 7d). The results show that the designed catalyst exhibits a high current density (1220.8 mA cm^−2^) and exceptional C2 product selectivity (81.4%) (Figure 7e), providing valuable insights into the large‐scale synthesis of C2 products via the CO_2_RR.

CO_2_RR involves a multistep reaction process of multi‐electron and proton transfer. Due to the weak bond energy of the intermediates *COOH and *CO, while the strong bond energy of C═O, SANIs electrocatalysts with multiple reaction active sites are required for synergistic catalysis, thereby enhancing the selectivity of the C2 product. Therefore, for CO_2_RR, electrocatalysts with diatoms‐ or multi‐atoms should be designed to improve the synergistic effect, thereby reducing the binding energy of the C═O bond to improve the catalytic selectivity.

### 4.4 SANIs Catalyst Applied in NOR

Applying SANIs catalysts in the NOR underscores their unique structural advantages and exceptional performance across diverse electrocatalytic reactions. Beyond the aforementioned reaction types, SANIs electrocatalysts exhibit considerable potential in the NOR. This discovery broadens the scope of SANIs catalyst applications and provides insights and directions for related fields. Nucleophiles, including alcohols, aldehydes, urea, glucose, and hydrazine, have increasingly become focal points for replacing the OER coupled with the HER for hydrogen production owing to their low theoretical potential and high‐value‐added products.^[^ ^80^ ^]^ Traditional OER processes necessitate high overpotentials and suffer from low energy‐conversion efficiencies, hindering the commercial development of green hydrogen. The NOR can mitigate the potential demand of the overall reaction and enhance the overall economic benefits through high‐value‐added oxidation products. Consequently, preparing SANIs electrocatalysts with high catalytic activity, stability, and selectivity for the NOR is vital. In this regard, Hui et al.^[^ ^37^ ^]^ synthesized PtCl_2_Au(111)/GDY catalysts using GDY as support and epitaxially grew Au quantum dots to anchor single Pt atoms. The catalyst exhibited high mass activity in methanol and ethanol oxidation reactions and maintained stable performance for 110 h without considerable decay. SANIs catalysts’ unique attributes, such as their atomically dispersed single‐atom sites, confined‐space effects, and interatomic interactions, contribute to their exceptional catalytic performance in the NOR. Furthermore, the application of SANIs catalysts in the NOR coupled with the HER can moderate reaction conditions. Traditional hydrogen production via water electrolysis typically requires high temperatures and pressures; however, incorporating SANIs catalysts can substantially reduce the reaction temperature and pressure, thereby enhancing reaction safety and operability. SANIs electrocatalysts hold immense potential for NOR–HER‐based hydrogen production. Further refinement of the catalyst structure, composition, and catalytic performance and stability can facilitate the commercialization of green hydrogen.

SANIs catalysts have shown remarkable advantages in applications such as water splitting, fuel cells, and CO_2_ reduction, etc. To maximize its development in the field of electrocatalysis, the feasibility and industrial‐level stability under industrial conditions should be considered. In addition, SANIs electrocatalysts with “island–sea synergistic” structure have unique advantages for electrocatalytic reactions with multiple reaction steps and paths due to active metal atoms with multiple coordination environments. In the following studies, different active sites should be loaded on SANIs for different reaction intermediates to improve the reaction rate and achieve selective catalysis.

## 5 Summary and Outlook

SANIs represent an innovative confined‐space SACs material with a distinctive structural design. This design effectively stabilizes single atoms, substantially mitigating their migration and aggregation. It elicits exceptional activity, stability, and selectivity across various electrocatalytic reactions. This review comprehensively elaborates on the recent advancements in SANIs electrocatalysts and their unique advantages compared to traditional SACs. Furthermore, we discuss the design strategy of SANIs catalysts in terms of supports and nano‐islands, providing invaluable insights into future catalyst designs. SANIs catalysts have demonstrated remarkable catalytic efficacy in various electrochemical reactions through their unique confined single‐atom coordination environment.

**Figure 8:**
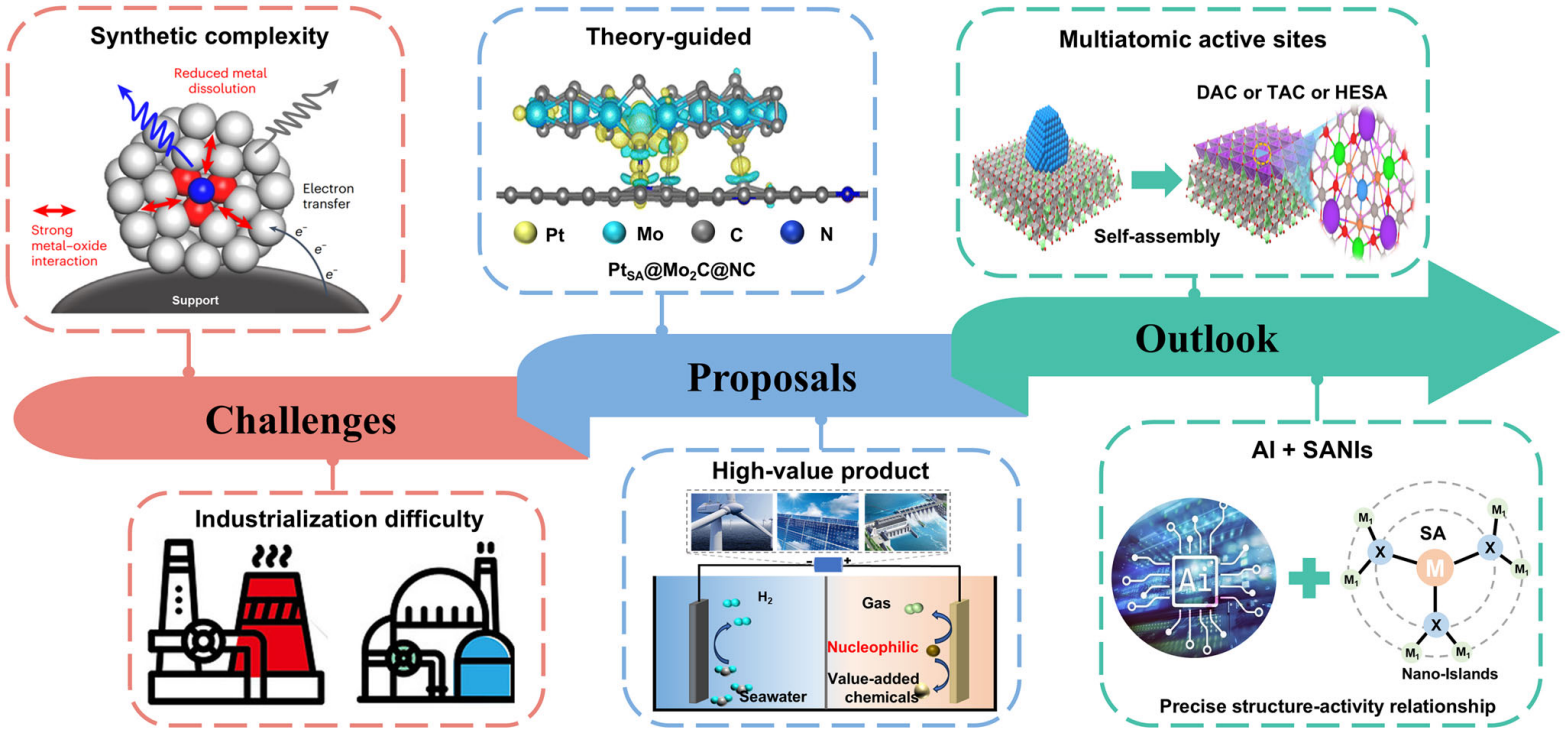
Challenges, prospects, and outlook of SANIs electrocatalysts.

Furthermore, we explore the interactions between the single atoms and nano‐islands or supports, such as MSIs, vacancy defects, and spatial confinement effects. These interactions are important in fine‐tuning the atomic coordination environment, thereby enhancing the catalytic performance of SANIs catalysts. SANIs electrocatalysts have alleviated the challenges of reduced activity and stability associated with single‐atom aggregation to an extent. However, they are still in the laboratory research phase, with a notable gap to be covered before large‐scale preparation and industrial application. To propel the development of SANIs electrocatalysts, we objectively discuss the challenges and opportunities associated with this promising technology (**Figure** **8**).

### 5.1 Synthesis Method of SANIs Electrocatalysts

Owing to the intricate SANIs structure, sophisticated and multistep synthesis strategies are required. Although SANIs catalysts have been successfully synthesized through wet chemistry, atomic deposition, and other techniques, harsh synthesis conditions and limited control over individual active sites pose notable challenges for large‐scale preparation (at the gram or even kilogram level). Consequently, reproducible, straightforward, and scalable preparation methods, such as 3D printing and ball milling, are required to advance SANIs catalysts in industrial catalysis. Furthermore, the material selection for SANIs electrocatalysts is diverse and fraught with opportunities and challenges. Thus, the heterogeneous‐interface effects between different materials and the potential interactions between single metal atoms and supports or nano‐islands must be considered before material selection. By integrating machine learning with theoretical calculations, the advanced screening and prediction of SANIs catalysts with high performance and selectivity can theoretically guide the efficient synthesis of SANIs electrocatalysts. Future research should focus on achieving the directional loading of single metal atoms onto nano‐islands and precisely controlling the coordination microenvironment of single atoms.

### 5.2 Characterization of SANIs Electrocatalysts

Currently, the accepted comprehensive characterization methods for SACs include spherical aberration‐corrected TEM (physical state), X‐ray absorption spectroscopy (coordination environment), infrared spectroscopy (electronic properties), and theoretical calculations (atomic structure). However, SANIs catalysts constitute a multiphase system involving various phase states, such as metal atoms, nano‐islands, supports, and reactants, with complex interactions and electron transfer processes occurring at multiple interfaces. Furthermore, the active center of SANIs catalysts undergoes dynamic changes during reactions, potentially involving evolutions from single atoms to single‐atom clusters. Thus, developing characterization and probe techniques with high energy and high spatiotemporal resolutions is crucial to understanding the dynamic reaction processes of SANIs catalysts and enhancing the selectivity of complex catalytic pathways. Notably, given the diversity of single‐atom coordination microenvironments, using coordination microenvironment descriptors can promote the innovation and development of accurate and controllable synthesis methods for SANIs.

### 5.3 Industrial Application of SANIs Electrocatalysts

Although reports on applying SANIs electrocatalysts in heterogeneous catalysis exist, studies achieving industrial current densities remain relatively scarce. Moreover, it is challenging to scale up SANIs electrocatalysts for industrial catalytic processes while maintaining catalytic activity, stability, and selectivity on a laboratory scale. Thus, it is important to design multitype active sites and optimize the coordination microenvironment of single atoms to improve the intrinsic activity of SANIs electrocatalysts. Furthermore, extending SANIs to dual‐atom nano‐islands or high‐entropy SANIs is a promising design strategy that can enhance the synergistic effect of polymetallic active sites, boost catalytic activity, and achieve high‐precision selective catalysis. Notably, SANIs electrocatalysts must operate in complex industrial environments, such as high temperatures (50–80 °C) and highly corrosive electrolytes. Thus, to develop more practical SANIs electrocatalysts, SANIs electrocatalysts must be optimized to withstand highly corrosive environments. Simultaneously, SANIs electrocatalysts should be extended to more electrochemical reactions, such as the NOR and electrochemical syntheses, to produce higher‐value‐added products.

### 5.4 Reaction Process and Mechanism of SANIs Electrocatalysts

With the rapid development of computer technology, theoretical simulation has emerged as a powerful tool for investigating electrocatalytic reaction processes and mechanisms at the atomic scale.^[^ ^81^ ^]^ For instance, DFT calculations based on quantum mechanics can be employed to investigate the adsorption and reaction energy barriers between the active site of SANIs electrocatalysts and different reaction intermediates. This concept is based on known electronic‐structure models to theoretically validate the mechanism underlying their excellent electrocatalytic performance. Molecular dynamics simulation can dynamically describe the electron motion state during electrocatalysis; this is invaluable when determining the reaction mechanism of electrocatalysts. The diversity of active sites in traditional heterogeneous catalysts leads to an unclear structure–activity relation, rendering rational design challenging. However, SANIs electrocatalysts possess a clear active‐site coordination microenvironment, which can serve as a minimal primitive of the catalyst to obtain the required artificial intelligence (AI) label data for the intrinsic activity of the involved single atom. Thus, with the support of AI‐assisted machine learning and big data, constructing an accurate structure–activity relation database will provide robust support for the rational design of SANIs catalysts. In the future, applying AI‐assisted design to dual‐atom nano‐islands and even high‐entropy SANIs catalysts might become possible, thereby propelling the development of SANIs electrocatalysts.

## Conflict of Interest

The authors declare no conflict of interest.
